# Viral Strategies and Cellular Countermeasures That Regulate mRNA Access to the Translation Apparatus: Second Edition

**DOI:** 10.3390/v18091021

**Published:** 2026-09-15

**Authors:** Christopher U. T. Hellen

**Affiliations:** Department of Cell Biology, State University of New York Downstate Health Sciences University, Brooklyn, NY 11203, USA; christopher.hellen@downstate.edu

## 1. Introduction

Viruses depend on the cellular translation apparatus, and the translation process is consequently a focus for competing mechanisms to promote and to restrict viral translation. In this Special Issue, leading experts presented and reviewed insights into the means used by viruses to subvert the cellular translation apparatus to support viral translation and to maximize utilization of their genomic coding potential, the processes employed by host cells to restrict viral translation, and the mechanisms that viruses have developed to counter these cellular defenses.

Eukaryotic cellular mRNAs conventionally have a 5′-terminal 7-methylguanosine ‘cap’, a 3′ poly(A) tail and 5′ and 3′ untranslated regions (UTRs) flanking an open reading frame (ORF). The canonical initiation process [1] begins with formation of a 43S preinitiation complex by binding of eukaryotic initiation factor (eIF) 1, eIF1A, eIF3 and eIF2•GTP/initiator tRNA to the 40S ribosomal subunit. The 43S complex is recruited to the capped 5′-terminal end of mRNA by eIF4F, which comprises eIF4E (cap-binding protein), eIF4A (RNA helicase) and eIF4G (scaffolding protein) subunits. The 43S complex scans the 5′UTR until it encounters the first AUG triplet in a favorable nucleotide context (ideally, AccAUGG), which serves as the initiation codon. Scanning is impaired by secondary structure in the 5′UTR. Base-pairing between the initiation codon and the anticodon of initiator tRNA in the peptidyl (P) site of the 40S subunit leads to 48S complex formation. eIF5 and eIF5B then induce hydrolysis of eIF2-bound GTP, reorientation of initiator tRNA, release of eIFs and joining of a 60S ribosomal subunit to form a translation-competent 80S ribosome.

Many of these stages in initiation are negatively regulated in response to viral infection, for example by phosphorylation of eIF2 (which impairs recruitment of initiator tRNA), by sequestration of the 5′ end of viral mRNAs by interferon-induced proteins with tetratricopeptide repeats (IFITs) or by sequestration of eIF4E by the 4E-binding protein (4EBP). Moreover, many viral mRNAs have characteristics that are not compatible with efficient initiation by the canonical mechanism. For example, many are not capped, and their 5′-termini are instead covalently bound to a genome-linked protein (VPg). The noncoding 5′-terminal regions of VPg-linked and other viral RNAs form highly structured elements that impair ribosomal attachment and scanning.

## 2. Non-Canonical Mechanisms of Translation Initiation

Such viruses have developed alternative non-canonical mechanisms of translation initiation—for example cap-independent initiation mediated by a 3′ cap-independent translation enhancer (3′ CITE) and cap- and end-independent initiation mediated by an internal ribosomal entry site (IRES). Lu et al. reviewed recent developments regarding the structure and mechanism of action of these elements [2]. IRESs are classified into six major groups and have been identified in a growing number of families of positive-sense RNA viruses, primarily *Picornaviridae*, *Flaviviridae* and *Dicistroviridae* [3]. CITES typically occur in the 3′UTRs of positive-sense plant RNA viruses, and they have been assigned to seven structural classes. They engage in functional interactions that involve eIF4E, eIF4G and ribosomal subunits, in addition to long-range interactions with sequences in the 5′UTR that facilitate cap-independent initiation. Advances in knowledge of the structures of IRESs and CITES have increased understanding of how their interactions with canonical eIFs and IRES *trans*-acting factors (ITAFs) mediate cap- and/or end-independent initiation of translation. Intriguingly, Lu et al. [2] noted topological conservation in the tertiary structures of elements of the type 2 encephalomyocarditis virus (EMCV) IRES and the type 3 hepatitis A virus IRES that both interact with the central domain of eIF4G, pointing to underlying structural and mechanistic similarities between these classes of IRES and, more recently, between them and type 1 IRESs [4].

The sequences, structures and mechanism of action of type 6 IRESs are unlike those of IRES types 1, 2, 3 and 5. They had until recently been thought to be restricted to members of *Dicistroviridae*, exemplified by Cricket paralysis virus (CrPV), and to be structurally homogeneous, consisting of nested pseudoknots PK2 and PK3 appended to a 3′-terminal pseudoknot 1 (PK1) that mimics the anticodon stem-loop of tRNAs. PK1 underlies the ability of these IRESs to mediate factor-independent initiation of translation by binding directly to the ribosomal aminoacyl (A) site before undergoing eEF2-mediated translocation to the P site. However, recent analyses of metagenomic datasets have identified type 6 IRESs in members of *Narnaviridae* (order *Picornavirales*) and *Tombusviridae* (order *Tolivirales*), and revealed unexpected structural diversity [5,6]. Chapagain et al. [7] analyzed the activity of the *Halastavi árva* virus (HalV) IRES, which comprises only two pseudoknots (PK1 and PK2), enters the P site of 80S ribosomes directly and bypasses the initial eEF2-dependent translocation step [8]. They demonstrated its activity in insect cells and cell-free lysates, and reported that it supports translation and replication in the context of a heterologous (CrPV) viral replicon. Notably, its activity is upregulated during CrPV infection and as a result of ER stress. These conditions may simply increase the availability of vacant 80S ribosomes, but an intriguing possibility is that virus- or stress-induced changes in the translation apparatus that remain to be identified are responsible for activating the function of type 6 IRESs.

## 3. Exploitation of Non-Canonical Translation Mechanisms to Maximize Utilization of the Coding Potential of Viral Genomes

The size and structure of many viral genomes are constrained by (i) the canonical mechanism of translation initiation in eukaryotes, which restricts translation to a single ORF, (ii) the potentially lethal combination in many viruses of a lack of proofreading coupled with a low fidelity of replication, and (iii) the packaging limit of virions. Viruses overcome some of these limitations to maximize utilization of their genomic coding potential by exploiting alternative non-canonical translation mechanisms that enable ribosomes to access overlapping reading frames, additional ORFs or 3′-terminal extensions of the principal ORF. Hellen [9] discussed mechanisms employed by picornaviruses to expand utilization of the genomic coding potential that include internal initiation, leaky scanning, programmed ribosomal frame-shifting and reinitiation. Stop codon readthrough has been characterized in bacterial, plant and animal viruses, and Kamoshita [10] discussed the determinants and molecular mechanism of this process in animal viruses.

The ability of IRESs to promote end-independent initiation provides two mechanisms for the translation of a second ORF in positive-sense viral RNAs [9]. First, the ORFs may be translated independently, enabling the timing and stoichiometry of their translation to be differentially regulated. Thus in members of *Picornaviridae*, a type 1 -related IRES separates structural and nonstructural protein-encoding ORFs in the *Picodicistrovirus* genus, and a type 4 IRES separates the polyprotein-encoding ORF1 from an accessory ORF in some members of the *Megrivirus* genus. Secondly, initiation mediated by type 1 and type 2 IRESs is often ‘leaky’, and many picornaviruses exploit or have the potential to exploit leaky scanning to initiate translation of an accessory protein encoded by an out-of-frame ORF. Picornaviruses also modulate elongation to enable translation of alternative ORFs. Elegant studies (e.g., [11]) determined that a stimulatory element in cardiovirus genomes binds to the cardioviral 2A protein to activate programmed −1 ribosomal frame-shifting. By diverting ribosomes, this protein-dependent riboswitch also downregulates nonstructural protein synthesis late in infection. Termination/recycling may also be modulated, for example on megrivirus genomes that contain a second conserved ORF overlapping or adjacent to the ORF1 termination codon. Some of these genomes contain a ‘termination upstream ribosomal binding site’ (TURBS) analogous to an element that promotes termination-reinitiation in caliciviruses, whereas other picornaviruses have been hypothesized to exploit a novel mechanism of termination-reinitiation [9].

Stop codon readthrough involves decoding of a stop codon by a near-cognate tRNA, allowing the synthesis of a C-terminally extended polypeptide, and it occurs on translation of mRNAs from various animal, plant and bacterial viruses. In a comprehensive review, Kamoshita [10] described the Beier and Grimm classification of viral stop codon readthrough (based on stop codon identity and the sequence of flanking nucleotides) and provided a detailed molecular account of translation elongation and termination in mammals, focusing on how kinetic proof-reading and ribosomal structure ensure the fidelity of these processes. Stop codon readthrough has been intensively investigated for six species of animal virus from *Retroviridae*, *Togaviridae*, *Reoviridae* and *Dicistroviridae* families, for example leading to translation of the *pro* (protease) and *pol* (polymerase) genes in members of *Retroviridae*, and of the nsP4 viral replicase in alphaviruses (family *Togaviridae*). Stop codon readthrough occurs when near-cognate tRNAs overcome proofreading steps to decode the stop codon and thus enable translation of downstream coding sequences. This process is influenced positively and on occasion negatively by mRNA sequence (including the identity of the stop codon, proximal and distal sequences flanking the stop codon and downstream structures, by the sequence/structure and post-transcriptional modification of the P-site tRNA, by ribosomal components and by *trans*-acting factors. As a result, the efficiency of stop codon readthrough ranges from 2 to 15%, much higher than the misincorporation rate during elongation.

The levels of isoacceptor tRNAs corresponding to different synonymous codons vary between organisms, cell types and tissues. They determine the rate at which these codons are decoded and thus the rate at which mRNAs are translated in different cells. *A priori*, the codon composition of viral genomes would be expected to have evolved to match the tRNA supply in host cells, but as discussed by Ventoso [12], the codon usage of human viruses often deviates from ideality. Many viral mRNAs are enriched in A/U-ending codons, which are associated with reduced translation and stability. The failure to fully adapt may reflect host-mediated mutational pressure, including the activities of the cellular nucleic acid editing enzymes APOBEC3 (apolipoprotein-B mRNA-editing complex 3) and ADAR-1 (adenosine deaminase acting on ribonucleic acid 1), and the fact that CpG and UpA dinucleotides are pathogen-associated molecular patterns that activate the zinc finger antiviral protein and RNAse L. Although the codon usage of many viral mRNAs is suboptimal, they are nevertheless efficiently translated in infected cells, likely because shut-off of host translation minimizes competition for cytoplasmic tRNAs.

## 4. Cellular Antiviral Stress Responses and Viral Mechanisms to Counter Host Defenses

Viral infection induces cellular stress responses that impair viral replication, promote cell survival and if necessary, trigger cell death. These processes include the unfolded protein response (UPR), the integrated stress response (ISR), apoptosis, and antiviral innate immune responses. They affect translation by targeting the activity of translation factors, impairing access of the translation apparatus to viral mRNAs and promoting their degradation. Viruses counter these host defenses by encoding proteins that disrupt these responses [13,14] and by the presence in their mRNAs of sequence and structural elements that enable them to bypass host-imposed restrictions on translation [2] and to co-opt cellular proteins and mechanisms that safeguard genome integrity [15].

The 3′poly(A) tails of viral mRNAs enhance translation and stability, and they are a target for 3′deadenylation by the cellular CCR4-NOT deadenylase complex, which is commonly the initiating step in degradation of eukaryotic mRNAs. Wilusz [15] summarized different approaches used by viruses to counter this process and to maintain the integrity of their poly(A) tails. One approach involves the binding of proteins to sequence motifs in the 3′UTR, epitomized by binding of the HUR/ELAV1 protein to the 3′UTRs of alphavirus mRNAs. Various cytoplasmic nucleotidyl transferases such as TENT4 add non-templated nucleotides to the 3′-end of cellular mRNAs (including non-adenosine residues, which reduce the rate of deadenylation). TENT4 with its co-factor ZCCHC14 have been coopted by various viruses to stabilize the poly(A) tail. Recent studies identified additional mechanisms for poly(A) tail elongation, each promoted by specific viral recognition sequences and mediated by poly(A) polymerase gamma, poly(A) polymerase alpha or TENT4 in conjunction with another cofactor, ZCCHC2 [16]. A third mode of stabilization involves blocking access of deadenylases to the 3′-end of the mRNA by binding of proteins to the poly(A) tail and to the poly(A)-binding protein, or by sequestration of the poly(A) tail in triplex structures.

Badu and colleagues [13] reviewed interconnected cellular stress responses to viral infection, and describe how they are subverted by different flaviviruses to enable replication. For example, the UPR is an endoplasmic reticulum (ER) stress program that is regulated by ER-embedded sensors that activate three downstream signaling pathways. Flavivirus nonstructural proteins engage directly with UPR factors to modulate these pathways or to modify the temporal order of their activation, thereby enhancing cellular survival (and extending the timeframe for viral replication), promoting viral protein synthesis and ensuring the correct folding of viral proteins. The integrated stress response (ISR) is orchestrated by the kinases PERK (an ER sensor that also acts within the UPR pathway), protein kinase R (PKR), general control non-depressible 2 (GCN2) and heme-regulated inhibitor (HRI). They converge on the alpha subunit of eIF2, phosphorylating it and ultimately impairing translation initiation. Flaviviruses activate the ISR via PERK and PKR, but also subvert it to support viral replication. As noted by Badu et al. [13], many molecular details of how flavivirus proteins suppress these antiviral responses remain to be determined.

By contrast, the basis for evasion by poxviruses of PKR is understood in detail, and is a paradigm for the evolutionary ‘arms race’ between host cells that evolve mechanisms to abrogate viral translation, and viruses as they evolve mechanisms to evade cellular antiviral defenses [14]. PKR is a pattern recognition receptor that recognizes viral dsRNA and phosphorylates eIF2’s alpha subunit, leading to the inhibition of mRNA translation. Poxviruses encode two PKR antagonists that target different steps in the PKR activation pathway: K3 (encoded by the K3L gene) is a pseudo-substrate inhibitor that prevents the interaction of PKR with eIF2α, and E3 (encoded by E3L) is a dsRNA-binding protein that prevents dsRNA-binding by and consequent activation of PKR. PKR has undergone rapid evolution, so that PKR from different organisms show very different levels of inhibition/resistance to K3 from a given virus. The K3L gene is consequently a determinant of host range. Here, Park and colleagues [14] extended studies of the molecular basis for the contrasting inhibition profiles exerted by vaccinia virus K3 (which inhibits bovine PKR strongly, but is a weak inhibitor of ovine PKR) and the sheeppox virus K3 ortholog (which has the opposite activity profile). Experiments involved domain exchanges and site-directed mutagenesis of bovine and ovine PKR, complementing a panel of PKR moieties from a range of organisms, and they yielded insights into the molecular basis for the host-specific inhibition of a key antiviral protein by viral antagonists.

Many viruses prevent translation of innate immune effectors by targeting components of the cellular translation apparatus and/or by inducing selective degradation of cellular mRNAs, resulting in shut off of host protein synthesis. There has been rapid progress in elucidating the mode of action of the Nsp1 protein encoded by Severe acute respiratory syndrome coronavirus 2 (SARS-CoV-2), a member of the betacoronavirus genus of *Coronaviridae*. SARS-CoV-2 Nsp1 occludes the entrance of the mRNA-binding channel of the 40S subunit to prevent access of mRNA, and induces endonucleolytic cleavage of host mRNAs. However, the mechanism(s) by which Nsp1 moieties encoded by other alpha- and beta-coronaviruses induce shut-off of cellular translation is poorly understood. Ohkami and colleagues [17] analyzed the host gene suppressive mechanism employed by bovine coronavirus (BCoV), a betacoronavirus, and determined that BCoV Nsp1 binds to the ribosomal 40S subunit and induces strong shut-off in a manner dependent on ribosomal association. However, they did not detect endonucleoytic cleavage of host mRNA, emphasizing variation in mechanisms of Nsp1-mediated regulation of gene expression.

## 5. Summary

The papers in this Special Issue focused on recent advances in characterizing mechanisms that viruses have developed to exploit the translation apparatus in host cells, to restrict translation of cellular mRNAs in infected cells and to counter cellular defenses that restrict viral translation. This collection of papers emphasized the remarkable diversity of these mechanisms, and highlighted the many important questions that remain to be resolved.

## Data Availability

No new data were created or analyzed in this study. Data sharing is not applicable to this article.
